# Explaining Human Recreational Use of ‘pesticides’: The Neurotoxin Regulation Model of Substance Use vs. the Hijack Model and Implications for Age and Sex Differences in Drug Consumption

**DOI:** 10.3389/fpsyt.2013.00142

**Published:** 2013-11-05

**Authors:** Edward H. Hagen, Casey J. Roulette, Roger J. Sullivan

**Affiliations:** ^1^Department of Anthropology, Washington State University, Vancouver, WA, USA; ^2^Department of Anthropology, California State University, Sacramento, CA, USA

**Keywords:** pharmacophagy, zoopharmacognosy, drug reward, evolution, self-medication, evolutionary medicine

## Abstract

Most globally popular drugs are plant neurotoxins or their close chemical analogs. These compounds evolved to deter, not reward or reinforce, consumption. Moreover, they reliably activate virtually all toxin defense mechanisms, and are thus correctly identified by human neurophysiology as toxins. Acute drug toxicity must therefore play a more central role in drug use theory. We accordingly challenge the popular idea that the rewarding and reinforcing properties of drugs “hijack” the brain, and propose instead that the brain evolved to carefully regulate neurotoxin consumption to minimize fitness costs and maximize fitness benefits. This perspective provides a compelling explanation for the dramatic changes in substance use that occur during the transition from childhood to adulthood, and for pervasive sex differences in substance use: because nicotine and many other plant neurotoxins are teratogenic, children, and to a lesser extent women of childbearing age, evolved to avoid ingesting them. However, during the course of human evolution many adolescents and adults reaped net benefits from regulated intake of plant neurotoxins.

The mesolimbic dopamine system (MDS)^1^ plays a key, though still not fully understood, role in the ability of laboratory animals to learn an association between a stimulus, such as a tone, and a natural reward, such as sugar water, and to approach and consume the reward (1–6). Drugs of abuse have neurobiological and behavioral effects that closely resemble the effects of sugar and other natural rewards, activating the MDS and producing approach and consummatory behavior, positive feelings, and the learning of cues that predict drug availability. Drugs *are* rewards (7). Moreover, drugs and sugar are chemically similar: both are small organic molecules that act as ligands for various receptors. In fact, fermentation converts 1 glucose molecule into 2 ethanol molecules (and 2 CO_2_ molecules), and ethanol contains more calories per gram than glucose (7 vs. 4), underscoring the comparability of a natural reward and a drug. On what basis, then, do neurobiologists classify drug reward as abnormal and food reward as normal?

## 1 The Hijack Hypothesis

Numerous, highly cited articles that review the neurobiology of drug use employ similar metaphors to distinguish natural rewards from drugs: natural rewards “activate” the MDS, whereas drugs “hijack,” “usurp,” “co-opt,” or artificially stimulate it [e.g., Ref. (7–15)]. Kelley and Berridge [(9), p. 3306], for instance, open their review with:

Addictive drugs act on brain reward systems, although the brain evolved to respond not to drugs but to natural rewards, such as food and sex. Appropriate responses to natural rewards were evolutionarily important for survival, reproduction, and fitness. In a quirk of evolutionary fate, humans discovered how to stimulate this system artificially with drugs.

In another review, Hyman [(11), p. 1414] leads into a section titled “A Hijacking of Neural Systems Related to the Pursuit of Rewards” with:

[A]ddiction represents a pathological usurpation of the neural mechanisms of learning and memory that under normal circumstances serve to shape survival behaviors related to the pursuit of rewards and the cues that predict them.

On the evolutionary novelty of drug dependence, Wise [(8), p. 27] is perhaps most explicit:

Addiction is quite a recent phenomenon, largely dependent upon the controlled use of fire (smoking), hypodermic syringes (intravenous injection), and the cork and bottle (storage and transportation of alcohol). Thus, while brain dopamine is activated by most drugs of abuse, the drugs have undergone mostly human selection for their ability to activate the system; the system has not undergone natural selection because of its sensitivity to the drugs.

We refer to these arguments as the “hijack hypothesis.” We recognize, on the one hand, that this is a metaphor invoked by drug researchers to help explain the effects of drugs of abuse on the brain. On the other hand, its frequent appearance in prominent review articles suggests that drug abuse researchers consider it to provide a fundamental distinction between addictive substances and food. This distinction is based on the following Darwinian propositions: the MDS evolved to enhance access to some substances, like sugar, that increased fitness^2^; these are termed “natural rewards.” It did not evolve to respond to known drugs of abuse because these did not increase fitness and because repeated consumption of such substances is an evolutionary novelty^3^.

Unfortunately, most drug researchers do not seem to regard the hijack hypothesis as a hypothesis. Instead, it is treated as an axiom or truism that requires little supporting evidence. The most important point of our commentary is that the evolutionary premises of the hijack hypothesis are *empirically testable*.

Previous work has criticized the hijack hypothesis on a number of grounds (see, for instance, articles in this special issue). In particular, although laboratory studies demonstrate that animals will self-administrate most drugs of abuse, these studies rarely provide the animals with alternative rewarding choices. In studies that do provide a rewarding alternative, such as sweetened water or social interactions (as in the famous Rat Park experiment), most animals choose the alternative, not the drug, undermining the claim that drugs hijack decision-making machinery (19).

Here we briefly summarize our previous critique of the hijack hypothesis’ evolutionary premises (20–22). It is important to emphasize that we only critique these premises, *not* the evidence on the neurobiological mechanisms involved in drug use nor the various interpretations of dopamine function. We then sketch an evolutionary alternative to the hijack hypothesis: the *neurotoxin regulation* hypothesis. We conclude by considering age and sex differences in substance use in light of both hypotheses.

A caveat: neurobiological theory of drug use usually contrasts initial seeking and use with longer-term phenomena such as drug tolerance and addiction. We focus on initial drug seeking and use for several reasons: there are a small number of simple information-processing models of initial drug seeking and use, often dubbed “reward models.” Current research on drug tolerance and addiction, in contrast, lacks a similarly concise, well-accepted conceptual framework [for a review of various theories of addiction, see Ref. (23)]. Moreover, tolerance and addiction are generally attributed, in part, to complex changes in neurobiology induced by long-term drug exposure. It is difficult to evaluate which changes are due to the effects of drugs and which to the nervous system’s attempt to adapt to drug exposure, complicating an evolutionary analysis.

## 2 Most Drugs are Plant Defensive Chemicals or Close Chemical Analogs

Terrestrial plants and animals appeared ∼400 million years ago. Animals evolved to exploit plant tissues and energy stores, and in response, plants evolved numerous defenses, including toxins. These toxins appear in high concentrations in some organs, like leaves, that are critical for plant growth, survival, and reproduction, and in low concentration in other organs, like ripe fruits, that evolved to be consumed by herbivores to aid seed dispersal, which is beneficial for the plant.

Plant drugs, such as caffeine, nicotine, cocaine, and THC, belong to a subcategory of toxins that evolved to interfere with neuronal signaling in herbivores. Depending on the toxin, this includes interference with: (1) neurotransmitter synthesis, storage, release, binding, and re-uptake, (2) receptor activation and function, and (3) key enzymes involved in signal transduction (24). Plant drugs therefore did evolve to “hijack” herbivore nervous systems, but for an effect that is precisely the opposite of the hijack hypothesis: to deter, not reward, or reinforce, plant consumption. (We prefer describing these effects as “interference” rather than “hijacking.”)

Plant toxins have had a profound influence on the evolution of herbivore neurophysiology, resulting in: (1) numerous chemosensors including bitter taste receptors, (2) detoxification mechanisms including cytochrome P450 and other enzymes, (3) cellular membrane carrier proteins for toxin transport, including ATP-binding cassette proteins, and (4) aversive learning mechanisms that permit selective feeding on less toxic tissues (25, 26). Many herbivore defensive proteins are expressed in the blood-brain barrier and the brain itself, including in humans (27–30), indicating the fitness advantages of protecting the CNS specifically from plant neurotoxins and other xenobiotics.

From an herbivore’s perspective, then, the value of a plant substance usually comprises the benefits of useable macronutrients (carbohydrates, fats, and proteins) minus the costs of toxin exposure.

### 2.1 Benefits of toxin consumption in non-human animals

Although exposure to plant toxins is ordinarily costly for herbivores, herbivores have also evolved to exploit plant toxins for herbivore benefit, which often involves prophylactic or therapeutic effects against pathogens, i.e., self-medication (also known as pharmacophagy or zoopharmacognosy) (31–43). Originally proposed as a primate behavior, evidence for self-medication is now available from diverse non-human species, including fruit flies (40, 41), ants (44), moths (39), butterflies (45, 46), honeybees (47, 48), birds (42), sheep (49), goats (50), and Neanderthals (51). In many of these studies (but not all), animals increase toxin intake in response to infection. More generally, there is growing recognition that animal defenses against pathogens include not only immune system responses, but also behavioral responses, termed *behavioral immunity* or *non-immunological defense*, of which self-medication is one example (52, 53).

In summary, animals have been exposed to plant toxins, likely including those affecting the CNS, for hundreds of millions of years. Animals can also extract benefits from such exposure. Thus, the evolutionary premises of the hijack hypothesis – that, for humans, drug exposure is evolutionarily novel and has no fitness benefits – are questionable and cannot be accepted without considerable further evidence.

### 2.2 Nicotine as a model drug

In what follows we will often rely on studies of tobacco and nicotine for the following reasons: first, nicotine is globally popular and highly addictive. Second, it is a plant drug, and therefore belongs to the category of substances that most animals were regularly exposed to during their evolution. Third, it is not out of the question that humans have chewed or smoked various psychoactive plants for hundreds of thousands of years, just as tobacco is consumed today. Fourth, the role of nicotine as a plant defensive chemical is well-documented (54, 55). And fifth, there is extensive research on nicotine.

We will also draw on the extensive research on pharmaceuticals and pesticides because often these are derived from plant toxins (e.g., nicotine, which has therapeutic applications and is also widely used as a pesticide), chemically resemble plant toxins, or have neurophysiological effects analogous to plant toxins. Data on them will therefore help us illuminate neurophysiological responses to plant toxins.

### 2.3 Nicotine toxicity

Although neurobiology emphasizes the rewarding properties of nicotine, nicotine is an extremely potent neurotoxin. In humans, the lethal dose of nicotine is ∼10 mg in children and 30–60 mg in adults, a toxicity comparable to hydrogen cyanide (56). Death can occur within 5 min after consumption of concentrated nicotine insecticides (57). A single cigarette typically contains 10–20 mg of nicotine, but much of it is burned; smokers thus absorb 0.5–2 mg per cigarette, and users of smokeless tobacco about twice this much (58).

Despite the evolutionary novelty of human exposure to nicotine^4^, nicotine activates most known human toxin defense mechanisms, such as bitter taste receptors in the mouth and gut (62), bitter taste pathways in the peripheral nervous system (63), xenobiotic-sensing nuclear receptors (64), xenobiotic-metabolizing enzymes (58), aversion circuitry in the CNS (65), and conditioned taste avoidance (66).

In individuals not habituated to nicotine, 0.6 mg (one “light” cigarette) can induce sweating, nausea, dizziness, coldness of hands, palpitations, headache, and upset stomach (67); 4–8 mg often produces serious symptoms, including burning sensations in the mouth and throat, profuse salivation, vomiting, abdominal pain, and diarrhea (57).

Human neurophysiology thus correctly identifies nicotine as a dangerous toxin and generates appropriate avoidance and expulsion responses. Because nicotine is not thought to be directly responsible for the chronic diseases caused by smoking (68) [cf. Ref. (69)], its toxicity plays little role in research on tobacco use. More generally, although drug researchers have long recognized that drugs are toxins and have aversive effects, and that drug toxicity and aversiveness is at odds with drug reward [for reviews, see Ref. (70–72)], this insight has had little influence on drug use theory (72). In the framework we develop here, however, drug toxicity plays a central role.

## 3 The Neurotoxin Regulation Hypothesis

Herbivores and omnivores, including humans, obtain substantial macronutrients from plants. Plant choice in non-human animals is heavily influenced by toxin concentration, which appears to be assessed by chemosensors in, e.g., the mouth and gut, followed by conditioned learning and social learning (e.g., observing mother’s plant choices) (73, 74). Complete avoidance of plant toxins is not an option, however. Mammalian herbivores cap the daily amount of ingested plant toxins by modulating intake to accommodate changes in the dietary concentration of toxins. They are able to do this even for toxins that are, for them, evolutionarily novel (75). It appears that herbivores regulate the dose of plant toxins to keep blood concentrations below a critical level [Ref. (76), and references therein]. At the same time, because plant toxins can provide fitness benefits, regulatory mechanisms should not, and could not, completely eliminate exposure to plant toxins but instead balance dose-dependent costs vs. benefits, and adjust intake accordingly [Ref. (74, 77, 78), and references therein].

In our view, drug toxicity poses two major challenges to any theory of drug use. First, why do humans ignore cues of toxicity, like bitter taste and nausea, to regularly and deliberately consume non-trivial doses of potentially lethal substances that provide essentially no macronutrients? Second, given that humans do consume such substances, how and why does human neurophysiology successfully meter their intake? The hijack hypothesis seems to imply that drug consumption is regulated, at least in part, by the same mechanism that regulates consumption of sugar and other foods. Humans consume tens-to-hundreds of grams of sugar and other carbohydrates per meal. Typical doses of recreational drugs, on the other hand, are tiny – on the order of milligrams or tens of milligrams – and are not far below a lethal dose (79); yet overdoses and death are relatively rare^5^. We find it surprising that the inadvertent triggering of a mechanism that evolved to reward and reinforce intake of large quantities of macronutrients results in the precisely metered intake of minute quantities of neurotoxins.

We therefore propose that the brain might not accidentally reward or reinforce consumption of nicotine and other addictive drugs, as the hijack model proposes, nor generate purely aversive reactions, as drug toxicity would suggest, but instead has evolved specialized mechanisms to precisely *regulate* drug consumption to minimize costs and maximize benefits [Ref. (22) cf. Ref. (81)].

A neurotoxin regulation mechanism would only evolve if, in fact, there were fitness benefits to neurotoxin consumption. We have proposed numerous potential benefits of psychoactive drug use, including enhancement of attention, memory and other aspects of cognition and physiology, and redressing nutrient deficiencies and neurotransmitter dysregulation (20–22). Later, we sketch another possible benefit involving attraction of mates and other social partners.

Our principal hypothesis, however, has been that human consumption of plant neurotoxins helps prevent or treat infection by parasites with nervous systems, i.e., macroparasites such as helminths [see also Ref. (82)], similar to the self-medication observed in many other animals species. Helminth parasites have been an important selection pressure in vertebrate and mammalian evolution, and in human evolution specifically (83, 84). Over one third of the global population remains infected by them (85). Helminths are often able to evade the immune system (86, 87), so chemotherapeutic intervention is frequently necessary to clear infections. There is increasing evidence that some non-human animals consume plant toxins specifically to prevent or treat helminth infections (49, 50) [but see Ref. (88)].

Intriguingly, three of the world’s most popular psychoactive drugs – nicotine, arecoline (from betel-nut) and THC – are effective against helminths and other macroparasites; to this day some farmers and veterinarians deworm animals with nicotine or arecoline (89–98). Some helminth species have a larval stage that migrates through the lung (84), which perhaps was a selection pressure specifically to smoke neurotoxic plants^6^.

An evolved mechanism to self-medicate with psychoactive substances should up-regulate consumption and down-regulate elimination of such substances in response to infection and/or infection risk. There are intriguing hints that infection risk and immune system signals do just that^7^. The “proinflammatory hypothesis of drug abuse” has emerged from growing evidence of immune involvement in drug reinforcement (102–104). Opioids, for instance, perhaps acting as xenobiotic-associated molecular patterns, activate toll-like receptor 4 (TLR4) signaling (important for pathogen recognition and immune activation), which surprisingly reinforces opium consumption via the mesolimbic dopamine reward pathway (105). Especially intriguing is direct evidence that the immune system modulates intake of the psychoactive drug ethanol (106, 107). One genome-wide association study found that smoking behavior might be regulated by IL-15, which is involved in immune signaling (108). Such results indicate an intimate relationship between psychoactive drug use and immunity, and, importantly, that central immune signals can modulate drug consumption.

Down-regulation of drug metabolism during infections would increase blood concentrations of potentially therapeutic agents. Infection and inflammation are indeed associated with a broad down-regulation of xenobiotic-metabolizing enzymes and transporters in humans and laboratory animals (albeit with complications for CYP2A6, which metabolizes nicotine), which often results in a pronounced increase in plasma concentrations of various drugs. This well-documented but poorly understood phenomenon (109) could also be evidence for a self-medication mechanism.

Although it might not be intuitive to reconceptualize recreational drug use as a means to prevent or treat macroparasite infections (chemoprophylaxis and chemotherapy, respectively), we point out that prior to the discovery of sodium’s role in body fluid homeostasis, our evolved appetite for salt was utterly mysterious.

There is considerable evidence that nicotine intake is tightly controlled. If nicotine were purely rewarding or reinforcing, then lethal nicotine overdoses among adult tobacco users should be common. Instead, they are extremely rare (79). Behaviorally, cigarette smokers appear to titrate nicotine, altering their smoking behavior in response to changes in nicotine content so as to maintain a relatively constant blood concentration of nicotine (110). Both facts support the existence of a regulatory mechanism.

The putative regulatory mechanism might involve the MDS, which seems to play a central role in weighing the costs of behaviors, not just benefits. A subpopulation of dopamine neurons in the MDS is excited by aversive stimuli and cues that predict aversive stimuli (111–113). There is even one report that bitter taste receptors are expressed in the rat MDS (30). Given the anatomical proximity of the targets of aversion- and reward-related dopamine, their interaction could be the neurophysiological basis for weighing costs against benefits (2, 114). Interestingly, the MDS appears to be involved in the neurophysiological system that evolved to regulate intake of small quantities of sodium (115, 116). We envision the hypothesized neurotoxin regulation mechanism to be somewhat analogous to the salt appetite regulation mechanism in that it would employ numerous peripheral and central chemosensors and feedback circuits to precisely meter intake of milligrams of environmental chemicals.

Unlike the sodium regulation mechanism, the putative neurotoxin regulation mechanism must titrate a diverse range of compounds, many of which would be evolutionary novel for the organism: plants are constantly evolving new chemical defenses, and both plants and animals migrate. Currently, it is not possible to fully explain how a limited number of toxin defense proteins, which would be the foundation of a regulatory mechanism, selectively bind to a large range of chemically diverse toxins. Understanding the relationship between the physiochemical properties of a toxin molecule and its biological activity – its structure-activity relationship – is a dynamic and challenging area of research [e.g., Ref. (117)]. Part of the answer is that most toxins belong to one of a smaller group of chemical families, such as fatty acids, peptides, amino acids, amines, amides, azacycloalkanes, N-heterocyclic compounds, ureas, thioureas, carbamides, esters, lactones, carbonyl compounds, phenols, crown ethers, terpenoids, secoiridoids, alkaloids, glycosides, flavonoids, and steroids (118). Molecules belonging to the same family tend to share chemical properties^8^. Thus, binding regions of defensive proteins might be specific for classes of compounds.

In addition, chemically diverse toxins can interfere with the same signaling pathway (e.g., nicotine, a small organic molecule, and botulinum toxin, a protein, both interfere with cholinergic signaling). We speculate that a neurotoxin regulation mechanism might be able to detect interference with neural signaling pathways, and modulate intake accordingly. We also speculate that individual learning plays an important role in the neurotoxin regulation mechanism. Given exposure to a novel neurotoxin with unknown costs and benefits, a user should first ingest minute quantities, gradually increasing intake to optimize benefits vs. costs, which resembles patterns exhibited by laboratory animals and humans with extended access to drugs (119).

## 4 Age Differences in Drug Use: A Developmental Switch?

There are dramatic changes in substance use across the lifespan, which provide an opportunity to empirically test the hijack hypothesis against the neurotoxin regulation hypotheses. Do these changes reflect changes in vulnerability to hijacking? Or do they reflect age-related changes in the costs and benefits of exposure to plant toxins that should up- or down-regulate ingestion?

**Figure 1:**
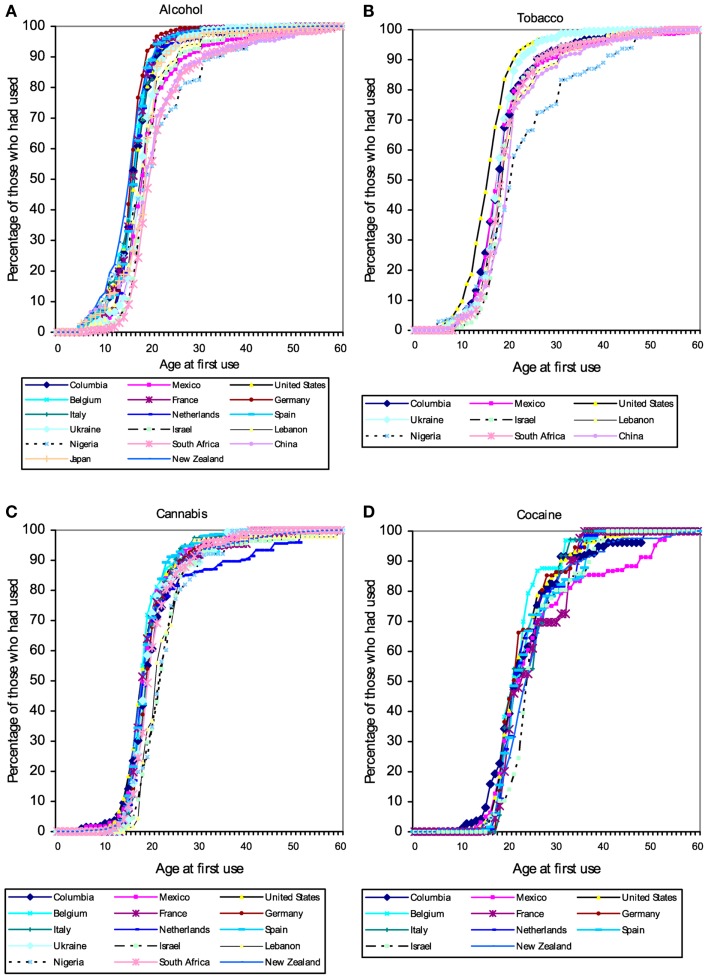
Cumulative distribution of self-reported age of first use of **(A)** alcohol, **(B)** tobacco, **(C)** cannabis, and **(D)** cocaine in a large (*N* = 85,052) cross-national sample of users of these substances from the World Mental Health Survey Initiative. These patterns suggest the existence of a developmental “switch” that flips during adolescence. Figures from Ref. (120).

Users of popular psychoactive substances report virtually no use prior to the age of 10 (with the partial exception of alcohol). Starting about the age of 12 there is a rapid increase in substance use, so that almost everyone who will ever use a substance has done so by age 20 (Figure 1). The pattern suggests the existence of a developmental “switch.”

**Figure 2:**
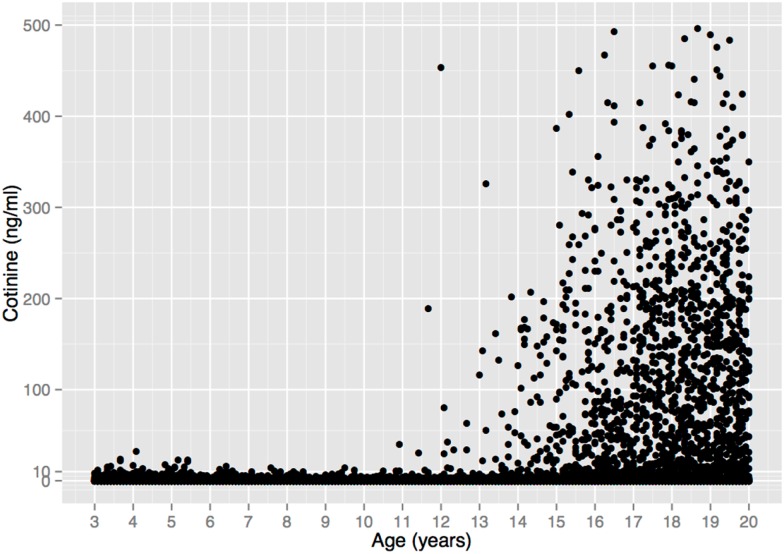
**Serum cotinine concentration vs. age in a sample of 18,382 children, adolescents, and young adults from the US National Health and Nutrition Examination Survey 1999–2010**. There is no unequivocal evidence of tobacco consumption prior to age 11. (6 Cotinine values >500 ng/ml were omitted for clarity, all from individuals >17 years old.) Data from Ref. (123).

The complete lack of child substance use seen in the self-report data in Figure 1 are corroborated by serum cotinine values from a nationally representative US sample (Figure 2). Cotinine, the primary metabolite of nicotine, is a reliable and widely used biomarker of exposure to tobacco, via either tobacco consumption or environmental tobacco smoke (ETS) (121). The cotinine concentration of a smoker is usually ∼100 ng/ml, whereas that of a non-smoking child living with smokers is usually <10 ng/ml (121, 122)^9^.

Figure 2 depicts 12 years of data (1999–2010) that include 5932 children ages 3–10 (123), 1111 of whom (19%) lived with a smoker, and thus presumably had easier access to tobacco. Among all children, 94.5% of the cotinine concentrations are ≤3 ng/ml and 99.4% are ≤10 ng/ml. These values are within the range of values seen in non-smokers exposed to ETS, e.g., from a smoking parent or other caregiver^10^. Only 33 children (0.6%) had cotinine values >10 ng/ml, and 6 (0.1%) had values >20 ng/ml, with the maximum value (32.4 ng/ml) occurring in a 4-year old. These values overlap with the values of smokers who haven’t smoked recently, but are still within the range of values that could result from heavy ETS, such as traveling in a car with a heavy smoker (121)^11^.

What explains the dramatic lack of child substance use, and the equally dramatic transition to substance use during adolescence?

### 4.1 The hijack model of children’s low-to-non-existent substance use

The hijack hypothesis predicts that anyone with a functional MDS, that is, anyone for whom sugar is rewarding or reinforcing, will be susceptible to tobacco and other drugs. The everyday experience that children enjoy sweets, and thus have a functional reward system, is confirmed by studies of: (1) diet across the lifespan that show that a substantial fraction of the daily energy intake for US children and adolescents comes from sugar added to beverages and foods (125, 126), and (2) reinforcement learning that find that although children and older adults do show “deficits” in some aspects of reward processing relative to younger adults, reward-based learning mechanisms are quite functional in children (127). The hijack hypothesis therefore predicts that, all else equal, children would consume drugs of abuse at rates similar to adolescents and adults, contrary to the evidence in Figure 1. On its own, the hijack hypothesis cannot explain the dramatic changes in substance use across the lifespan. Drug use researchers therefore typically invoke additional explanations.

An influential hypothesis attributes the onset of drug use to a transient “imbalance” between the MDS and the prefrontal cortex (PFC) that emerges in adolescence. The PFC is believed to be responsible for executive control functions such as self-regulation, abstract reasoning, deliberation, response inhibition, and planning ahead (128–130). According to the hypothesis, these functions manage or curb the rewarding and reinforcing signals from the MDS, including those generated by drug use. The key insight of this hypothesis is that the MDS and PFC have different developmental trajectories: the MDS is largely mature by adolescence but the PFC is still developing into early adulthood. It is thought that the still-maturing PFC cannot adequately control the heightened reward responsiveness stemming from the mature MDS, thus explaining why adolescents engage in risky behaviors, such as unprotected sex and drug use [e.g., Ref. (129–131), and references therein].

How does the imbalance model explain the lack of child drug use? To our knowledge, the proponents of this model have not explicitly discussed child drug use. However, the clear implication would seem to be that in children, prefrontal cortex circuits, and the MDS, though still developing, are “balanced,” so the PFC is able to successfully manage the MDS, explaining why children typically do not engage in risky behavior. Specifically, child enjoyment of drugs would be successfully overridden by the executive control circuits of the PFC. This requires that children know that drug use is risky. We guess that proponents of this model would argue that parents and others teach children about the dangers of drugs. In addition, parents and society impose restrictions on child access to drugs.

Restricted access to tobacco could explain low-to-non-existent child use. In the US, the sale of tobacco to minors is illegal in all 50 states. Moreover, the US spends over $500 million annually on tobacco control measures (132), which include mass media anti-tobacco campaigns; disseminating health warnings via, e.g., cigarette packages and advertising; enforcing bans on tobacco marketing; monitoring tobacco use; enforcing some smoke-free legislation; and providing some tobacco cessation health care programs. Tobacco taxes also deter use.

If such warnings and social restrictions account for low child substance use, then, according the hijack model, children should readily consume plant drugs when they are absent. Caffeine, a bitter-tasting plant toxin^12^, is a psychostimulant that strongly interacts with the central dopaminergic systems via antagonism of endogenous adenosine (135). Caffeine is added to numerous beverages marketed to children, which suggests that parents and society are not overly concerned about child caffeine consumption. In fact, the daily amount of caffeine consumed from soft drinks is similar in US children, adolescents, and adults (136). It is far from clear, however, that the rewarding properties of caffeine motivate child consumption of soft drinks. Soft drinks contain high levels of sugar and other sweeteners, and, compared to coffee, about 1/3 the concentration of caffeine. The rewarding properties of sugar and artificial sweeteners obviously play a major role in child consumption of soft drinks; the role of caffeine is unclear.

**Figure 3:**
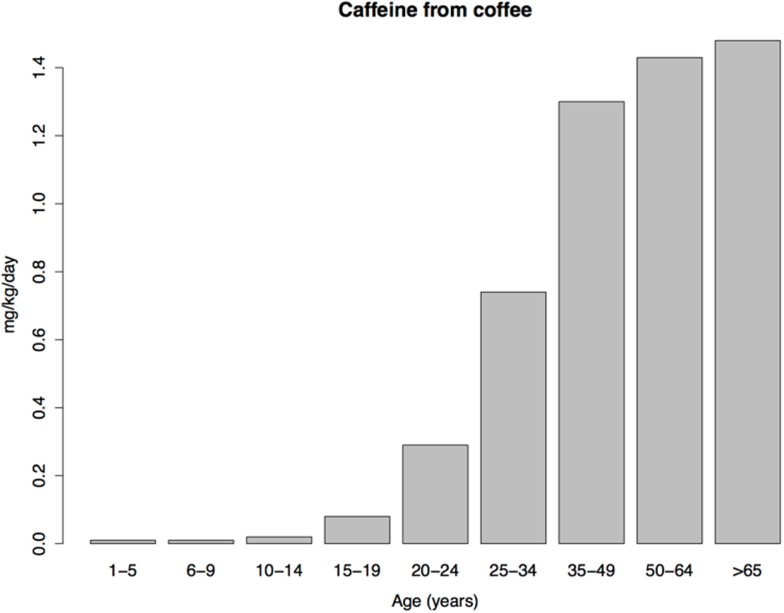
**Daily caffeine intake from coffee per kilogram of body weight in a sample of US caffeine consumers**. Data from Ref. (136).

Coffee consumption patterns should be informative because coffee contains a rewarding psychoactive substance (caffeine), does not necessarily contain sugar, and, unlike tobacco, is not subject to national or global efforts to control its consumption. Under the imbalance model, consumption of coffee should therefore be similar in children and adults. Yet coffee consumption is extremely low in US children, with a transition to adult levels occurring in adolescence and early adulthood (Figure 3), resembling the age pattern of tobacco use. The similar age patterns of tobacco and coffee consumption despite the profound difference in social restrictions on child access to tobacco vs. coffee, shows, at a minimum, that such restrictions play a smaller role in child drug consumption patterns than is commonly thought (we do not dispute their importance for adolescent and adult drug use prevalence). As we argue next, low drug use by children is probably better explained by child aversion to drugs.

### 4.2 The neurotoxin regulation model of children’s low-to-non-existent substance use

Because we take an evolutionary perspective, we consider the biological fitness consequences of plant toxin exposure to ancestral human children, who subsisted on wild foods. Virtually all wild plant foods, including fruits, contain toxins (137). A wild fruit contains substantial macronutrients, however, whose benefits can offset the cost of its toxins. A dried leaf of a neurotoxic plant, on the other hand, has virtually no macronutrients to offset the costs of its toxins. Thus, under the neurotoxin regulation hypothesis, consumption of the dried leaf would only occur when the benefits of toxin exposure outweigh the costs. Our basic premise, which we explain in detail next, is that during childhood the costs of toxin exposure outweigh the benefits, so that drug use is very low, but during adolescence the balance shifts, so that increased benefits outweigh diminishing costs, leading to substantial drug use.

#### 4.2.1 Age differences in the costs of neurotoxin exposure

We focus first on the fitness costs of exposure, and how these change during development. Due to differences in body mass alone, the cost of ingesting, e.g., 10 mg of nicotine, is much more dangerous to a 5-year old than a 15-year old. In addition, children have a considerably higher daily caloric requirement per kilogram of body mass: 2 year olds (a typical age of weaning in natural fertility populations) require about 80 kcal/kg/day, which decreases by young adulthood to about 40–50 kcal/kg/day (138, 139). This means that young children are eating almost twice as much food per kilogram of body mass as adults. Because ancestral humans relied on wild foods, higher caloric intake per unit mass implies potentially greater exposure to plant toxins per unit mass (depending on the “quality” of the diet, e.g., the mix of plant and animal foods).

This higher potential exposure has a number of implications. First, toxin defense pathways have limited capacities and can become saturated (140). Hence, consumption of a plant drug in addition to toxin-rich plant foods could cause toxin levels to reach dangerous levels. Second, toxin metabolism and elimination is energetically expensive (141), reducing energy available for, e.g., growth and immunity.

Disruption of development is perhaps the greatest cost of plant toxin exposure for children, though, because it can permanently impair functionality. Indeed, there is an entire discipline – teratology – devoted to investigating the role of environmental compounds in developmental disruption. Developmental toxicity is often distinct from systemic toxicity. For instance, low doses of some pesticides that cause little systemic toxicity nevertheless disrupt neural development, whereas near lethal doses of other pesticides have no discernible effect on neural development (142). The thalidomide tragedy provides another example: thalidomide was a sedative that was also effective against pregnancy sickness. Due to its low acute toxicity and the absence of teratogenic effects in rodents, it became quite popular in the 1960s until its severe teratogenic effects in humans – deformed limbs and organ defects in 20–30% of exposed infants – were recognized (143). The lack of teratogenicity in rodents might be due, in part, to their ability to rapidly metabolize and eliminate thalidomide, compared to much slower elimination in humans (144). These examples demonstrate that, for children, exposure to plant toxins can have costs above and beyond systemic toxicity, and that toxin metabolism is a key defense^13^.

Some popular plant drugs are indeed potent teratogens. Nicotine, for example, interferes with acetylcholine signaling, which has a unique trophic role in brain development, modulating the patterns of brain cell replication and differentiation, synaptic outgrowth, and architectural modeling. All phases of brain assembly, from the early embryonic stage through adolescence, are profoundly vulnerable to disruption by nicotine exposure (145, 146). Even child exposure to environmental tobacco smoke (cotinine concentrations of ∼1 vs. >100 ng/ml in tobacco users) is associated with deficits in neurodevelopment, intelligence, attention, and academic achievement (147, 148). Cholinergic signaling also plays an important role in non-neuronal cells, including those of the immune system, lungs, gut, and testes (149, 150), so nicotine and other cholinergic toxins could disrupt their development and function as well.

Consistent with these facts, there is considerable evidence for heightened toxin defenses during childhood. The best defense is to avoid ingesting toxic substances, and children reject many more foods than adults. Not surprisingly, vegetables and fruits are the most frequently rejected foods. There are two distinct, but closely related, psychological factors related to rejection of foods prior to ingestion: neophobia (rejection of novel foods), and “picky/fussy” eating (rejection of many foods, regardless of their novelty).

Neophobic food rejection occurs primarily due to visual cues. Foods that do not “look right” – green vegetables for example, or foods that resemble known bitter foods – are rejected without being placed in the mouth. Food neophobia is low at weaning, increases sharply as a child becomes more mobile (so parents would have less control over food choice), peaks between 2 and 6, and then decreases with age, becoming relatively stable in adulthood. Some studies show an inflection point at the onset of adolescence. The developmental trajectory of neophobia is widely interpreted to reflect an evolved defense against plant teratogens (151, 152). We see an important role for parental warnings in child ingestive behavior. Children should be averse to substances described by others as bitter or “bad.” Unlike the imbalance model, however, parental warnings about drugs are taken as cues of toxicity rather than an attempt to restrict access to desired substances.

Foods that are placed in the mouth are rejected primarily based on taste, especially bitter taste (though sometimes on texture) (152). Taste is responsible for evaluating the nutritious content of food and preventing the ingestion of toxic substances (see Box 1 for discussion of bitter taste physiology and genetics). Detection thresholds for bitter compounds are extremely low, in some cases as low as nano- or micro-molar concentrations (153), whereas those for sucrose are about 1000× higher (154).

Box 1 **Bitter taste physiology and genetics.**

To understand the strengths and limitations of the evidence for age and sex differences in taste, especially in bitter taste (toxin detection), it is helpful to know a bit about taste physiology and genetics, and the history of taste research. Taste receptor cells are each tuned to one of the five basic taste modalities: sweet and umami, which identify sugars and amino acids, respectively, two key nutrients; salty, which helps ensure proper electrolyte balance; and sour and bitter, which detect toxins. Taste buds, which are distributed across the tongue and palate epithelium, comprise 50–150 taste receptor cells. Circumvallate papillae are located at the back of the tongue and contain thousands of taste buds; foliate papillae are located along the back edge of the tongue and contain a dozen to hundreds of taste buds; fungiform papillae are located in the front two-thirds of the tongue and contain one or a few taste buds (155).

In the early 1930s it was discovered that the ability to taste the bitter compound phenylthiocarbamide (PTC) was inherited in a nearly Mendelian fashion (156, 157), with PTC taste blindness due to a recessive “non-taster” allele at a single locus having a population frequency of about 50% (158). Thus, about 25% of the population are homozygous for the non-taster allele and are non-tasters, and about 75% possesses at least one copy of the dominant taster allele and are “tasters.” The high frequency of the non-tasting allele implies balancing selection (e.g., heterozygote advantage), but the selective factor remains unknown.

An enormous body of research has explored variation in ability to taste PTC and a related substance, propylthiouracil (PROP), that has methodological advantages over PTC. One important finding is that PROP is intensely bitter for a subset of tasters, termed “supertasters,” and only mildly bitter for other tasters. Although it is tempting to conclude that supertasters are those who are homozygous for the taster allele, genotype, number of fungiform papillae (FP), and perhaps other factors make separate contributions to perceived PROP bitterness (159).

In the early 2000s, the genes for bitter, sweet, and umami taste receptors were identified. Whereas only 3 genes are involved in sweet and umami taste (T1R1, T1R2, and T1R3), about 25 functional genes code for human bitter taste receptors (the T2R family). This makes sense because the chemical diversity of toxins vastly exceeds that of macronutrients. Some bitter taste receptors respond to as many as 1/3 of known bitter compounds, whereas others respond to only a few; many bitter compounds active multiple receptors (118).

The gene responsible for the bimodal taste distribution of PTC/PROP was finally identified as one of the bitter taste receptors, T2R38, with tasters being homozygous or heterozygous for the PAV allele, and non-tasters being homozygous for the AVI allele (158). Thus, although human bitter taste is mediated by 25 taste receptors – all with allelic variation, all whose phenotypic expression is undoubtedly modified by other genes and environmental factors, and which, as a group, respond to thousands of compounds – most research on human bitter taste is based on the taste response to PTC or PROP, which is largely (but not entirely) mediated by two alleles of a single bitter taste receptor with a distinctive pattern of balancing selection. [Quinine, which activates 9 T2R receptors (118), is another bitter taste stimulant used in many studies.]

A further complication is that both T2R38 genotype and PROP phenotype predict intensity of non-bitter tastants, such as NaCl (salty), sucrose (sweet), and citric acid (sour). And even after controlling for T2R38 genotype, number of fungiform papillae, and nonoral sensory standards, PROP bitterness predicts intensity of other tastants (159). This means, among other things, that inter-individual differences in the intensity of toxicity signals inferred from T2R genotype or PROP phenotype might also predict differences in the intensity of nutrient signals, with potentially complex effects on ingestion.

Children 7–8 years old have a higher density of taste buds on the tip of the tongue than adults, and density is positively related to taste sensitivity. This density decreases to the adult level by about 9–10, with developmental changes complete by about age 11–12 (160). Children are indeed more sensitive to the bitter taste of PROP than adults, and are up to twice as likely to be supertasters (161), with the transition to adult sensitivity seeming to occur in adolescence [Ref. (162, 163), and references therein]. This age dependence might be greatest in T2R38 heterozygotes (163). (See Box 1). High bitter taste sensitivity leads to reduced consumption of bitter vegetables (e.g., cabbage, broccoli, asparagus, and spinach), especially in children (161).

All common recreational plant drugs, including nicotine, taste bitter. Ethanol has both bitter and sweet taste components (164). Thus, taste receptors properly recognize most popular psychoactive drugs as toxic. Given children’s heightened neophobia, bitter sensitivity, and pickiness, they probably find most psychoactive drugs to be especially unpalatable^14^.

The second major toxin defense is neutralization and elimination of ingested toxic substances, often via metabolism in the liver. As a percentage of body mass, liver volume is about 60% bigger in young children than in adults (165). Child drug clearance rates are very low at birth but reach adult levels by about 1 year, and then surpass adult levels, perhaps due to the increased relative size of the liver and/or higher expression of xenobiotic-metabolizing enzymes (166)^15^.

**Figure 4:**
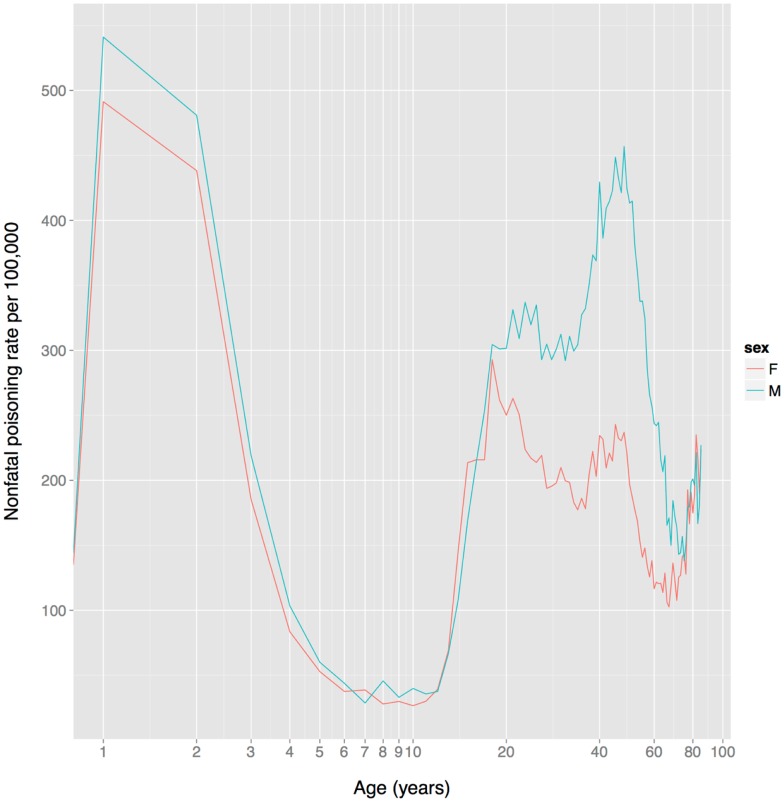
**US unintentional non-fatal poisoning rate vs. age, 2001–2011**. There is a dramatic decline in rates during childhood, a dramatic increase during adolescence, and the female (but not male) rate declines steadily during women’s reproductive years (about age 18–38). The x-axis is on a log scale to improve display of the lower age range. Data from Centers for Disease Control and Prevention (168).

Based on non-fatal poisoning data from the US, child toxin avoidance and metabolism mechanisms seem to work well: poisoning rates are highest in 1–2 year olds and then drop rapidly from 2 to 6, remaining low until adolescence (Figure 4), a pattern that mirrors the ontogeny of neophobia. Of course, this pattern might reflect other factors, such as increasingly tight restrictions on access to toxic substances and/or improved training of children with age. Poisoning rates increase sharply at the onset of adolescence, paralleling reduced neophobia and the onset of substance use.

The apparent existence of heightened toxin defenses in children strongly suggests that, for much of human evolution, the costs of exposure to plant toxins during childhood have been high, but diminished as brain and other organ development neared completion, i.e., in adolescence. Although ancestral human children could not completely avoid plant toxins, we propose that during childhood the costs of consuming plant substances with high levels of neurotoxins but low levels of macronutrients almost always outweighed the benefits. The result is that children’s heightened toxin defense mechanisms usually prevent drug ingestion, which explains children’s virtually non-existent drug use.

#### 4.2.2 Increases in fitness benefits across development

**Figure 5:**
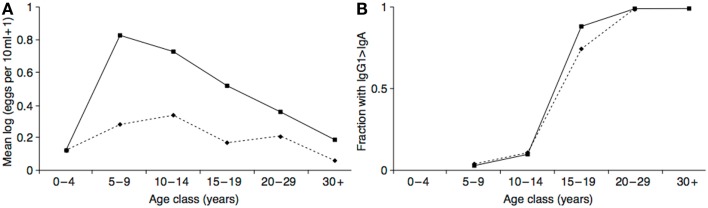
**(A)** Intensity of *Schistosoma haematobium* infection in two Zimbabwean populations with high transmission rate (solid line) and low transmission rate (dashed line). **(B)** The resulting antibody switch. The y-axis represents fractions of the two populations with high levels of anti-soluble egg antigen specific IgG1 relative to IgA (as absorbance at 492 nm). Reproduced from Ref. (170).

The hypothesized neurotoxin regulation mechanism functions to minimize the costs of exposure, and maximize the benefits. In addition to the evidence that the costs of neurotoxin exposure were diminishing in adolescence, there is also evidence that the putative anti-parasite benefits were increasing. In populations with endemic helminth infections (which presumably include ancestral human populations), individuals are born without infections but acquire them as they age. At the same time, they gradually acquire protective immunity. As a likely result of these two processes, infection levels peak in middle childhood or adolescence in many populations, with the age of the peak dependent on the parasite transmission rate and the rate at which individuals become immune (a higher transmission rate leads to an earlier, more intense peak) (169–171). See Figure 5A. This peak could have selected for a predisposition to initiate drug use at this time to maximize prophylactic or therapeutic benefits. Intriguingly, in populations with endemic *Schistosoma haematobium* infection the immune system itself appears to undergo an age-related antibody switch. Theoretical and empirical results suggest this reflects a transition from an early non-protective response based on exposure to eggs to a later protective response stimulated by the death of adult worms (172) (Figure 5B). If the immune system response to a helminth infection exhibits a switch-like transition in adolescence, then so, too, might behavioral defenses, such as self-medication.

Attracting mates is another possible benefit for adolescents but not children. Sexually selected signals, such as peacock tails and bird songs, are widespread in nature and usually emerge at the end of the juvenile period to advertise sexual maturity and mate quality to the opposite sex (173, 174). Similarly, conspicuous psychoactive substance use, which under the hypothesis cannot begin until the risk of teratogenesis has abated, would be a reliable cue or signal that a developmental milestone had been achieved, such as maturation of the nervous system and perhaps gonads or other organs^16^. Such a signal might attract mates (175) and other social partners because a developmentally mature individual would be able to provide them greater benefits. A reliable cue or signal of developmental maturity would be especially important in populations, such as most hunter-gatherers, that do not keep track of chronological age yet choose mates and social partners based on qualities that vary with age but are otherwise difficult to discern.

**Figure 6:**
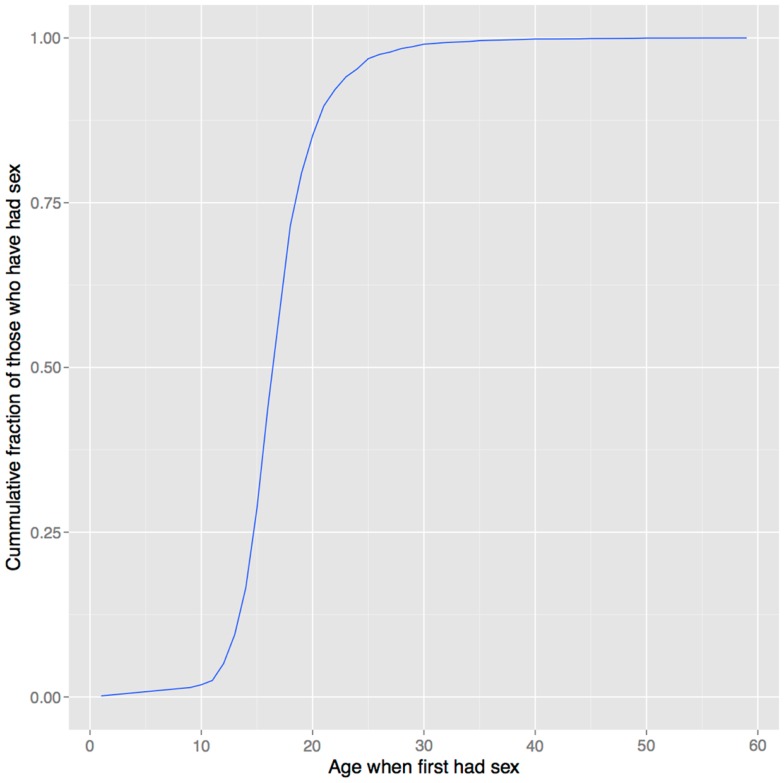
**Age of first sexual intercourse in NHANES data**. This cumulative distribution closely resembles that of age of first substance use seen in Figure 1. Data from Ref. (123).

In support of this hypothesis, the age of onset of sexual behavior closely parallels that of drug use (compare Figures 1 and 6). Smoking initiation is significantly influenced by perceived benefits like looking grown up (176); and in adolescents, perceived maturity, substance use, sexual behavioral, and prestige are all correlated (177, 178). Young smokers are also more risk taking and impulsive, traits that characterize males engaged in intrasexual competition (179–181), and engage in earlier sexual behavior (182), all of which suggest a link between substance use and mating. Indeed, higher mating effort is related to more smoking and more lenient attitudes toward drug use (183, 184). But, smoking has well-documented negative effects on female reproductive function [reviewed in Ref. (185)], and there is also evidence that it negatively impacts male reproduction, including erectile function (186, 187), so the signaling benefits to either sex would need to outweigh these costs^17^.

The neurotoxin regulation hypothesis, like the imbalance hypothesis, involves a cost-benefit analysis by children, but it differs from the imbalance hypothesis in a number of ways. First, we hypothesize that neurotoxin regulation involves specialized circuits and is not based solely on domain-general learning. Second, although these mechanisms take into account warnings from others, they rely heavily on bitter taste receptors and other chemosensors. Third, because the cost of ingesting too much neurotoxin vastly outweighs the cost of not ingesting enough, the mechanisms are biased against consumption. Fourth, the circuitry is well-developed in early childhood, although its functioning changes across development to reflect changes in the costs and benefits of neurotoxin exposure.

## 5 Sex Differences in Substance Use

**Figure 7:**
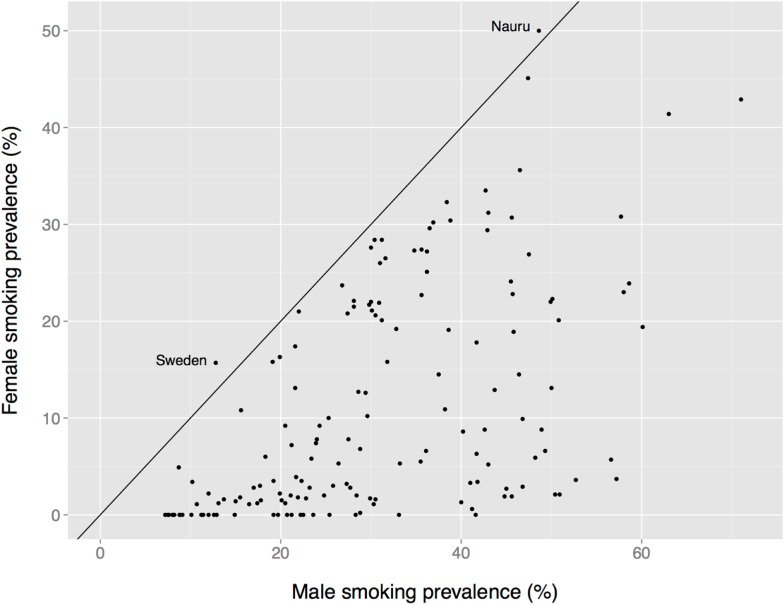
**Female vs. male smoking prevalence across nations**. The solid diagonal line represents equal prevalence. Values coded as “ <1%” set to 0. Data from the Tobacco Atlas (http://www.tobaccoatlas.org).

There is a global male bias in substance use among adults, albeit one that is less dramatic than the age bias, and that varies by nation, substance, age, birth cohort, and other factors. Male prevalence of smoking is almost always greater than female prevalence, for instance (Figure 7). The two exceptions in these data are Nauru (a small island in the south Pacific) and Sweden. The Swedish data are misleading, however, because the use of oral tobacco products is high among Swedish men but low among Swedish women. Thus, far more Swedish men use tobacco (40%) than do Swedish women (23%) (190).

The large cross-national WMHSI study (120) found that the odds ratio of women initiating use of alcohol, tobacco, cannabis, and cocaine use in any year of life, vs. men, ranged from 0 for tobacco use in Nigeria and 0.1 for cocaine use in Mexico and Columbia (large male biases) to a non-significant 0.8 for cannabis use in France (no sex bias). Although most of these ratios indicated a statistically significant male bias, when sex differences were examined by age there was clear evidence that, for some substances in some populations, they were narrowing in younger cohorts, especially for the legal drugs alcohol and tobacco, and in several cases there were no significant sex differences. In no case, though, were women significantly *more* likely to initiate use of these substances than men.

In contrast to the foregoing substances that either show a clear male bias, or no bias, there are some drugs that are more likely to be used by females of certain ages in some populations. For example, there is historical evidence for a female bias in the use of opioids in the late 19th and early 20th century US (191). In the US in recent years, adolescent girls (12–17) were more likely than adolescent boys to use alcohol and be non-medical users of psychotherapeutic drugs. Nevertheless, in the population as a whole, US men were more likely than women to be users of all categories of drugs, including psychotherapeutic drugs and alcohol (192, 193).

As with age bias, evidence of sex bias in substance use is based primarily on self-report, and could therefore reflect a sex bias in willingness to admit substance use rather than a sex bias in substance use itself. A comprehensive review of studies that compared self-reported smoking status to smoking status determined by cotinine levels (the biomarker of nicotine exposure) found that smoking was usually under-reported (194). A sex bias in under-reporting is less clear, however, with some studies finding that women under-report more than men, others finding that men under-report more than women, and culture seeming to play an important role. In two recent US studies, for instance, women’s self-reports were more accurate than men’s, or even overestimated cigarette consumption relative to men (195, 196), whereas in a large Korean study the ratios of cotinine-verified to self-reported smoking rates were 2.36 for women (substantial under-reporting) and 1.12 for men. Even so, cotinine-verified smoking rates were much higher in Korean men (50.0%) than women (13.9%) (197).

In short, unlike the nearly uniform global absence of child drug use (Figure 1), there is considerable heterogeneity in the prevalence of adult drug use by sex (e.g., Figure 7). This heterogeneity suggests that multiple factors differentially affect women’s and men’s drug use, perhaps including sex differences in access to drugs and sex differences in formal and informal social penalties and rewards for using drugs. At the same time, a greater prevalence among women appears to be the exception rather than the rule (e.g., Figure 7), which suggests that biological sex itself might play an important role in the decision to use, or not use, drugs.

### 5.1 The hijack model of sex differences in substance use

Women enjoy sugar, needless to say, and, in the US at least, eat as much of it as men (as a fraction of total calories) (198). This implies that there are no gross differences in food reward that would explain sex differences in substance use. On the other hand, there are numerous sex differences in motivation, reinforcement, and reward, and their underlying neural mechanisms that might. We will focus on the work of Becker and colleagues, who have written extensively on sex differences in the neurobiology of motivation and reward, and the implications for sex differences in drug use (199–202).

Becker and colleagues base their model on a plausible evolutionary account of sex differences in motivation rooted in parental investment theory. Parental investment is any investment, such as food or protection, by the parent in an individual offspring that increases the offspring’s survival (and hence reproduction) at the cost of the parent’s ability to invest in other offspring (203). Females in most animal species, including those that serve as models in drug research (e.g., rats, monkeys), invest more in offspring than males via, e.g., larger gamete size, internal gestation, and various forms of postnatal care, such as lactation. Male investment in offspring is minimal. As a consequence, female and male reproductive strategies diverge. Female fitness is largely constrained by access to the resources necessary to support high levels of parental investment, whereas male fitness is largely constrained by access to mates. Males therefore compete with other males for females, and females are choosey about mates (e.g., mating with males that exhibit higher genetic quality).

Becker et al. argue that, in rats, sex differences in parental investment have resulted in the evolution of sex differences in sexual and parenting motivation, and that some of these differences are shared with humans (200). In rats, male sexual motivation is constant, in line with a male strategy to maximize fitness by maximizing the number of mates. Male sexual motivation (but not mounting) is mediated by the MDS. Female sexual motivation, in contrast, depends on context and timing. Female rats are sexually receptive (estrus) for about 1 day of their 4–5 day estrous cycle. At this time, females “pace” copulations, and pregnancy is more likely to result when coital stimulation occurs at a particular rate. This appears to be a mechanism of female choice because dominant male rats contribute more intromissions and tend to give each female more ejaculations than subordinates (204). Female pacing is mediated by the MDS, and dopamine increases only when females anticipate receiving copulatory stimulation at their preferred rate of intromission.

There are also pronounced sex differences in parental motivation. Female rats exhibit a strong motivation to gain access to pups but males do not. There is some evidence that the MDS is involved in maternal motivation.

According to Becker et al., these sex differences in rat neurobiology emerge, in part, from the effects of gonadal hormones on the developing brain, particularly during the perinatal and peripubertal periods^18^. In addition, sex differences in gonadal hormones can result in sex differences in adult brain function.

Becker and Taylor [(200), p. 185] postulate that “Once sex differences in motivational circuits had evolved … there were unforeseen consequences that resulted in many other motivations systems being sexually dimorphic as well. Nowhere is this so striking as in drug addiction. Sex differences emerge in all phases of the addiction process including initiation and prevalence of use, patterns and levels of use, the progression to addiction, withdrawal, and relapse.” To more specifically link sex differences in drug use to sex differences in motivation, Becker and Taylor [(200), p. 178] argue that “motivation in females is modulated by gonadal hormones, and the female brain is more vulnerable to be co-opted by exogenous agents that induce constant activation (e.g., drugs of abuse) than are males.” And, “Sex differences in neural circuitry of attachment may spill over into other motivational systems too, including non-reproductive motivations for drugs. The development of strong attachments, and addictions or compulsive behaviors may occur through activation of the neural system that mediates maternal motivation; thus, females can become addicted to drugs more rapidly than males.”

Becker and colleagues cite a wealth of evidence from laboratory rats and humans that gonadal steroid hormones modulate drug-related behaviors, and that, by a number of measures, females in both species are more vulnerable to the effects of drugs (particularly cocaine). These sex-specific effects include more rapid progression from initial drug use to dependence in women, and more rapid acquisition of cocaine self-administration in female rats; greater stress-induced drug craving in women and female rats; and greater stress-induced reinstatement of drug use in women and female rats. Becker et al. (202) argue, further, that whereas men tend to use drugs for sensation seeking (positive reinforcement), women tend to use drugs to reduce stress or self-medicate psychological distress (negative reinforcement). Stressed or psychologically distressed individuals “enter into the downward spiral [toward addiction] already burdened with neurological changes that may promote their transition to addiction more rapidly.”

In summary, in rats the neurobiology of sexual and parental motivation and reward differs among the sexes and involves sex differences in the response of the MDS, and Becker et al. argue that these sex differences underlie sex differences in the animals’ responses to drugs of abuse. Many sex differences in human drug use are rooted in sex differences in human neurobiology that resemble those seen in rats. The upshot of most sex differences is that females are more vulnerable to the co-opting effects of drugs than males.

Becker and colleagues’ conclusion would seem to predict that the prevalence of drug use would be higher in women. Instead, for most drugs in most populations, the prevalence is higher, often much higher, in men (e.g., Figure 7). Becker et al. acknowledge higher male prevalence, and contend that it is a consequence of historical, cultural, and social factors. But the Becker et al. model is almost exclusively one of neurobiological sex differences and only briefly sketches what those historical and sociocultural factors might be: “Overall, availability of drugs coupled with dissatisfying social conditions, stress, anxiety, and depression tends to exacerbate drug abuse and addiction in women. While such conditions can also increase drug use in men, it is our hypothesis that on the average this happens more often in women” [Ref. (202), p. 5]. Maybe so, but of these factors, only “availability of drugs” has a plausible male bias that might explain the pervasive male bias in the prevalence of drug use. Like the imbalance model of drug use in children, then, the Becker et al. model puts the onus for lower prevalence in women on their socially restricted access to drugs.

### 5.2 The neurotoxin regulation model of sex differences in drug use

The neurotoxin regulation model of sex differences, like the Becker et al. model, is grounded in evolved sex differences in parental investment (and thus applies to adults, not children). According to the neurotoxin regulation model, the decision to ingest plant neurotoxins reflects an evolved calculus that weighs fitness benefits against costs. Because we take an evolutionary perspective, we consider the fitness benefits and costs of neurotoxin intake to ancestral men and women. Most of the fitness benefits and costs of regulated neurotoxin intake would probably have been similar for men and women, but women of childbearing age faced an additional cost: potential disruption of fetal and infant development.

Ancestral women had no access to highly reliable modern contraceptive technologies. Across contemporary hunter-gatherer societies that also lack such technologies, the median age at first birth is 19.25, the median weaning age is 2.5 years, the median interbirth interval is 3.2 years, and the median total fertility rate is 5.5 (205). Thus, for much of her late teens to her late thirties the median hunter-gatherer woman is pregnant or lactating.

Ancestral women’s plant ingestion therefore had a profound impact on the exposure of their fetuses and infants to plant toxins. Throughout gestation, for instance, maternal serum or urine cotinine levels correlate strongly with concentrations in fetal tissues and fluids such as cord blood, umbilical cord tissue, meconium, amniotic fluid, and placenta [Ref. (206), and references therein]. In infants breastfed by smoking mothers, cotinine concentrations in the urine are in the range of adult smokers (207). Fetal exposure to nicotine and other tobacco teratogens is associated with reduced academic achievement and intellectual abilities later in life (208).

Among contemporary hunter-gatherers, a median 21% of children die within the first year of life, and 45% within the first 15 years of life, rates similar to those seen in chimpanzees (205), which implies intense selection to protect offspring from harm. We therefore propose that, to maximize the fitness benefits from their high investment in offspring, women evolved to be more averse to toxins in their reproductive years, and to metabolize and eliminate them more rapidly. The tradeoffs of increased toxin defense included dietary restrictions and thus either reduced nutrient intake or increased search and processing times, and energy allocation to toxin metabolism at the expense of, e.g., activity levels and immunity.

Contemporary hunter-gatherer societies are characterized by a sexual division of labor, with men typically hunting or fishing, women typically gathering plant foods, and food widely shared among all group members (there is considerable variability, however, and women often hunt and men often gather) (205). If ancestral human societies were also characterized by a similar sexual division of labor involving foraging of plant vs. animal foods, then this could have been an additional factor for the evolution of sex differences in chemosensing and toxin defense.

Drugs of abuse activate most toxin defense mechanisms, including those governing intake such as bitter taste receptors and conditioned taste avoidance, and higher bitter sensitivity seems to reduce drug intake. For instance, T2R38 genotype predicts drug use in adults, with tasters consuming less than non-tasters. Ethanol tastes bitter, and beer and wine both contain additional bitter compounds (209–211). Alcohol intake is lowest in PAV homozygotes PAV/AVI heterozygotes (tasters); in one study (212) it was almost half that reported by AVI homozygotes (non-tasters) [reviewed in Ref. (213)]. A number of studies suggest that high bitter taste sensitivity in adults is also protective against nicotine dependence (214–219), albeit with some inconsistencies [e.g., Ref. (216)].

We therefore propose that heightened toxin defense in women (evidence for which we discuss next) results in their lower prevalence of drug use. We also propose that learning plays a key role: the effects of plant toxins on fetal and infant development are not completely predictable from their immediate physiological effects (e.g., bitter taste, nausea), so women should be attentive to information from others regarding the negative effects on offspring (or lack thereof) of ingesting particular plants, and adjust intake accordingly.

There is considerable evidence for sex differences in toxin disposition. Less clear is whether these differences are a consequences of greater toxin defense in women, particularly pregnant or lactating women, or instead are byproducts of, e.g., sex differences in physiology, such as body size and composition. Women have a higher percentage of body fat than men, for example, and lipophilic drugs, such as THC, are sequestered in fat tissue, which might account for some sex differences in response to THC (220). Sex differences in toxin disposition could even be due to sex differences in exogenous factors like diet. To give one example, grapefruit juice inhibits CYP3A4, an important drug metabolizing enzyme (221). If there were a sex difference in consumption of grapefruit juice, this could result in sex differences in the disposition of many drugs and toxins.

We will briefly review evidence that seems to suggest enhanced toxin defenses in women, while acknowledging considerable uncertainty in the interpretation of this evidence, that much evidence suggests no sex differences, and that some evidence points in the opposite direction. The challenge in resolving the nature of sex differences in toxin defense mechanisms is exacerbated by a lack of data on drug disposition in women, as pharmaceutical research often excluded women, particularly pregnant women, from clinical trials over concerns of possible drug teratogenicity.

We divide women’s lives into 3 distinct reproductive phases: a sexually active but pre-reproductive phase that starts in early-to-mid adolescence and ends at the age of first pregnancy; a reproductive phase involving alternating periods of pregnancy and lactation; and a post-reproductive phase that begins with menopause. We propose maximum sex differences in toxin defense during the reproductive phase, especially during pregnancy and lactation, and consequently maximum sex differences in the prevalence of substance use, but reduced sex differences in toxin defense in the pre- and post-reproductive phases, and consequently reduced sex differences in the prevalence of substance use.

Modern birth control technologies complicate interpretation of sex differences in drug use patterns and toxin defense mechanisms because for the first time in our evolutionary history women can indefinitely extend their sexually active pre-reproductive phase; reliably alternate psychoactive drug use with pregnancy and lactation; minimize breastfeeding by rapidly transitioning to infant formula; and sharply limit total fertility. In other words, during their reproductive years most modern women can, if they choose, use psychoactive drugs much of the time with little risk of fetal or infant exposure. Hormonal birth control introduces a further complication in that steroid hormones modulate xenobiotic metabolism (see below), and might also alter psychobehavioral toxin defense mechanisms.

#### 5.2.1 Heightened toxin detection in women

Women have more fungiform papillae and more taste buds than men and, according to most studies, are able to detect lower concentrations of PROP and are more likely to be supertasters (222). High bitter sensitivity, in turn, generally predicts reduced vegetable intake in both women and men [e.g., Ref. (223); for review, see Ref. (224)]. The major caveat is that research on bitter taste has been dominated by investigation of two compounds, PTC and PROP, which primarily activate a single bitter taste receptor, T2R38 (see Box 1); it is unknown whether sex differences in bitter taste sensitivity extend to a broad range of ecologically important substances and all bitter taste receptors.

#### 5.2.2 Heightened drug metabolism in women

Nicotine metabolism is accelerated in women (225). Nicotine and most other drugs are metabolized by liver cytochrome P450 enzymes. About a dozen of the 57 human P450 enzymes are primarily responsible for xenobiotic metabolism. Of the many factors influencing sex differences in drug disposition, there is widespread agreement that sex differences in hepatic enzyme activity play a major role.

The CYP3A family is the most abundant P450 in the liver, and is responsible for the metabolism of >50% of all commercial drugs. Most studies have found that women have about 20–30% higher CYP3A-mediated clearance, albeit with considerable variation across drugs and individuals (226, 227). Nuclear receptors (NR) are transcription factors that are activated by small lipophilic molecules, including plant toxins; NR, in turn, regulate the expression of many genes, including P450 enzymes. In rodents, sex differences in NR-regulated liver metabolism raise the possibility that the female liver is more efficient in neutralizing substances (228). In a study of 374 drug metabolizing and transporter genes in human liver tissue, sex differences in expression were found in 77 (21%). Of these, 58 (75%) had higher expression in women (229).

Other evidence suggests few sex differences in metabolism, or, for some substances, even a male bias. For instance, other than CYP3A, sex differences in the activities of most xenobiotic-metabolizing P450s are unresolved (227, 230). Some substrates of CYP1A2 and CYP2E1, which metabolize 4 and 2% of known commercial drugs, respectively (231), are more rapidly metabolized in men (232), and men appear to have greater hepatic expression of p-glycoprotein, an important drug transporter (230).

Based on FDA data, women suffer more prescription drug-related adverse events, and these events are of a more serious nature. This might indicate that women are *more* vulnerable to toxins, not less. It could also reflect the fact that most drug trials have involved men, and thus dosages are inappropriate for women due to smaller body size, differences in body composition, sex differences in pharmacokinetics, sex differences in pharmacodynamics (the effects of the drug), biased reporting, or perhaps that women more often use multiple drugs, increasing the risk of an adverse event (230). Because xenobiotics can both induce and inhibit P450 expression, sex differences in diet could also contribute to sex differences in adverse events.

In contrast to the FDA data on adverse events for prescription drugs, US non-fatal poisoning rates indicate that although there is no sex difference in adolescence, women have a markedly lower rate than men from age 20–75 (Figure 4). It is not clear whether this reflects heightened toxin defenses in women (including avoidance), or one or more of the many other factors influencing toxin ingestion, metabolism, physiological effects, and elimination.

#### 5.2.3 Pregnancy

During pregnancy, women have to meet increasing demands for macro and micronutrients while at the same time protecting their fetuses from plant teratogens, which exist in higher concentrations in the wild foods consumed by ancestral women than in the domesticated grains, vegetables, and fruits consumed by most women today. In addition, to accommodate a fetus expressing paternal genes, as well as changes in vulnerabilities to infection, there are substantial changes in maternal immunity.

Studies based on PTC/PROP find that bitter taste reaches a maximum in the first trimester of pregnancy, which would make women particular good “poison detectors” to protect the fetus from teratogens (233). A study using caffeine, however, which activates different T2Rs, found reduced bitter taste sensitivity in pregnant women vs. non-pregnant controls, an effect the authors interpret as functioning to increase variation in diet in order to increase weight during pregnancy (234).

Approximately 50–90% of pregnancies involve heightened food aversions, and up to 80% involve nausea and vomiting. This is puzzling given the increased micro- and macro-nutrient requirements of pregnancy. Yet nausea and vomiting in pregnancy (NVP), which tends to occur in the first trimester, is associated with positive pregnancy outcomes. Two complimentary adaptationist accounts of NVP have garnered widespread attention. One highlights aversions to meat, because meat is likely to harbor pathogens, and women are immunosuppressed in their first trimester (235, 236). The other, supporting the view we advance here, highlights aversions to toxic plants because these pose a risk to the developing fetus, especially during organogenesis (237, 238); for review, see (239). The two hypotheses broadly overlap, however, because microbial food-borne pathogens produce some of the most toxic substances known to science. Botulinum toxin, for instance (a cholinergic toxin like nicotine) has a lethal dose on the order of nanograms per kilogram of body mass (240).

Pregnancy-related aversions include drugs like alcohol, coffee, and tobacco (239, 241), and these aversions appear to reduce drug intake. Women smokers, for example, often reduce or cease smoking during pregnancy, and one important reason seems to be sensory aversions to tobacco smoke (242).

There are also pregnancy-related increases in drug metabolism. Nicotine metabolism, for instance, is accelerated in pregnancy (243). Activities of CYP3A4, CYP2C9, and CYP2D6, which together are responsible for the metabolism of >80% of commercial drugs, are increased several-fold during pregnancy. There is also evidence for increased activity of the phase II enzyme UGT1A4, as well as the drug transporters p-glycoprotein, OATP1B1, and OCT2. However, CYP1A2, which is responsible for the metabolism of about 4% of commercial drugs, is down-regulated. Pregnancy-related changes in activities of other P450 enzymes are equivocal, with some evidence for increased activity of CYP2B6.

Pregnancy hormones are obvious potential modulators of P450 enzymes. An *in vitro* study found that pregnancy-levels of estradiol enhanced CYP2A6, CYP2B6, and CYP3A4 expression, whereas progesterone induced CYP2A6 (minor), CYP2B6, CYP2C8, CYP3A4, and CYP3A5 expression (244). For reviews of drug disposition in pregnancy, see (231, 245–247).

#### 5.2.4 Menopause

If female toxin defenses are heightened to protect their fetuses and nursing infants, and if heightened defenses involve tradeoffs against other important functions and behaviors, then defenses should decrease to male levels post menopause. Consistent with this view, perceived bitterness of PROP remains relatively constant for women in their childbearing years, and then declines after menopause, whereas for men it shows a more gradual and steady decline from their 30s onward (233). Nicotine metabolism is accelerated in younger women compared to men, but is no different in menopausal and postmenopausal women than men (225). Data on the relationship between menopause and clearance of other drugs is conflicting, however. For example, some substrates of CYP3A4 are cleared less rapidly in menopausal and postmenopausal women, consistent with the hypothesis, but others show no difference (230).

#### 5.2.5 Summary of sex differences

In summary, there is enough evidence to propose (but not to conclude) that toxin defenses are heightened in women during their childbearing years; that the enhancement serves, at least in part, to protect the fetus and infant; that it reduces intake of drugs; and that it is partially responsible for the lower prevalence of drug use of women in their 20s and 30s. We speculate that the diminishing sex differences in use of some substances in younger cohorts might partially reflect the global fertility transition over the last several decades that involves increased use of birth control, later age at marriage, delay of first birth, and lower total fertility (248), all of which would allow women, especially younger women, to increase drug intake while limiting fetal and infant exposure. If the fertility transition is partly a consequence of reduced child mortality rates combined with women’s increasing economic importance in society and their need for increased education, and thus a reduced emphasis on their reproductive roles, this might also have reduced social disapproval of drug use in women relative to men, further increasing women’s access to, and willingness to use, drugs.

Finally, we suggest that, for the human data at least, the Becker et al. and neurotoxin regulation models are complimentary rather than contradictory. Whereas the neurotoxin regulation model of sex differences emphasizes female avoidance and elimination of plant toxins, the Becker et al. model primarily applies to women who have already chosen to use drugs, and who therefore differ from the general female population; given lower female prevalence of drug use, they might differ more than male drug users differ from the general male population. In addition, as Becker et al. emphasize, men and women often use drugs for different reasons, with women more often using drugs to alleviate stress or depression and men more often as a type of risk taking. It is not so surprising that people who use drugs for very different reasons would also exhibit important differences in many other facets of drug use.

## 6 Concluding Remarks

We have taken the idea that drugs hijack the brain and reframed it as a testable hypothesis. We then developed a testable alternative, the neurotoxin regulation hypothesis. In our view, the dramatic age and sex differences in drug use are better explained by age and sex differences in the costs vs. benefits of toxin exposure than by age and sex differences in reward, supporting the neurotoxin regulation hypothesis. The case is far from closed, however, and there is little reason to accept either hypothesis without considerable further research. The hijack hypothesis would be supported by finding that the child MDS generates a pro-drug motivation that is overridden by deliberations in the prefrontal cortex, perhaps in response to adult warnings, and/or is thwarted by socially restricted access to drugs. It would also be supported by finding that the sex differences in substance use are largely due to sex differences in social restrictions on drug use that are independent of childbearing, and not due to sex differences in a preference to consume drugs.

The neurotoxin regulation hypothesis would be supported by finding, instead, that child drug use is rare regardless of adult warnings or restrictions, there is no pro-drug use signal from the MDS to override, children are strongly averse to drugs, and, at least in ancestral populations, there were fitness benefits to drug use, which first exceeded the costs in adolescence. It would also be supported by finding that sex differences in drug use are partly a consequence of maternal toxin defense mechanisms that function to protect the fetus and infant. Increased drug use by pre- and post-reproductive women is not surprising, and recent diminishing of sex differences for some drugs in some populations might be linked to the fertility transition. Product engineering aimed at increasing women’s drug use, which includes adding sugar and flavorings to alcoholic beverages and cigarettes (249, 250), probably also plays a role in reducing sex differences.

Tobacco use and abuse of alcohol and other drugs are major contributors to global disease burden (251, 252). Many pharmacological treatments for drug abuse aim to reduce reward (253). Research on the treatment of substance abuse might benefit by also investigating pharmacological manipulation and enhancement of toxin defense mechanisms^19^.

## Conflict of Interest Statement

The authors declare that the research was conducted in the absence of any commercial or financial relationships that could be construed as a potential conflict of interest.

## Footnotes

^1^The MDS comprises dopamine (DA) neurons located in the midbrain structures of the ventral tegmental area (VTA) and substantia nigra pars compacta (SNc) that project to the nucleus accumbens (NAc) and dorsal striatum.

^2^By “fitness” we mean biological fitness – the average contribution to the gene pool of the next generation. For a review of fitness concepts, see Ref. (16).

^3^Some have recently argued that sugar is itself a potential substance of abuse [e.g., Ref. (17)], which again requires an appeal to evolutionary novelty, in this case the novelty of access to relatively unconstrained quantities of sugar [e.g., Ref. (18)].

^4^Whereas humans evolved in the Old World, all but one of the 60–70 tobacco species (genus *Nicotiana*) are native to the Americas or Oceania (one species is native to Africa). *N. tabacum*, the species of primary commercial importance, was domesticated by Native Americans within the last several thousand years, and spread to the Old World in the last few hundred years (59–61). Although nicotine is present in a number of plants native to the Old World, it is currently difficult to make the case that human ancestors were regularly exposed to nicotine.

^5^Drugs that are injected, and thus bypass peripheral chemosensors, are a partial exception. In the US in 2008, for example, there were about 1.9 million cocaine users and 5100 cocaine-related lethal poisonings, for an annual rate of 0.27%, which includes users who injected cocaine (CDC, NIH). A study of lethal cocaine-related deaths in Australia found that 86% involved cocaine injection and 81% also involved other drugs, mostly opioids, alcohol, and benzodiazepines (80). Thus, death from non-injected cocaine is rare.

^6^Contrary to Wise (8), there is archeological evidence for controlled use of fire c. 790 KYA – 1 MYA (99, 100), long before the evolution of *Homo sapiens*.

^7^The prevailing view is that the causal arrow usually points in the opposite direction, i.e., that drug abuse increases infection risk by impairing immunity (101).

^8^But it is not uncommon for small chemical changes to result in large changes in bioactivity.

^9^Distinguishing smokers from non-smokers exposed to ETS based on cotinine values alone is complicated by the many sources of variability, such as amount of nicotine exposure, time between last exposure and sample collection, and differences in nicotine and cotinine metabolism due to, e.g., common polymorphisms in the enzymes that metabolize them (primarily CYP2A6). A smoker who hadn’t smoked recently and a non-smoker exposed to heavy ETS might both have a cotinine concentration of 10 ng/ml, for instance. Estimates of the optimal serum cotinine concentration for distinguishing tobacco users from non-users exposed to ETS range from 3 to 15 ng/ml (121).

^10^In children living with smokers, the highest cotinine values in occurred in the younger children, suggesting physical proximity to a primary caregiver who is a smoker, or greater absorption and/or slower clearance of nicotine in younger children.

^11^A study of 42 non-smoking bar staff, for example, found a mean cotinine value of 9.28 ng/ml, with 3 individuals having values >20 ng/ml, and a maximum value of 31.3 ng/ml (124).

^12^There are two hypotheses for the evolution of caffeine, which is found in several plant species: herbivore defense, and allelopathy – inhibiting the germination of competing plants (133). Caffeine in nectar might have evolved to enhance pollinator memory (134).

^13^Metabolism can also convert non toxic compounds into toxins (e.g., thalidomide metabolites are biologically active), which probably illustrates the truism that no defense mechanism is perfect.

^14^Chocolate, a concoction of sugar and fermented cacao beans, sometimes with milk and other flavors, is an exception as it contains several psychoactive plant xanthine alkaloids, including theobromine and caffeine. However, the version popular with children is mostly sugar, fat, and protein.

^15^Pediatric drug clearance is the subject of continuing theoretical and empirical investigation, and it is possible that the increased drug clearance in children relative to adults is an artifact of linear scaling by weight instead of allometric scaling by 3/4 power or body surface area (167). Regardless, children achieve near-adult levels of drug clearance at an early age.

^16^It would also signal well-functioning detoxification mechanisms, of course.

^17^It is possible that such costs could increase the credibility of the signal, sensu (188, 189).

^18^Becker et al. aim to explain sex differences in drug use, not age differences. But their theory could explain the dramatic age differences discussed earlier. If drugs specifically hijacked the mechanisms underlying sexual behavior, parenting, or pair-bonding, then drug use would only occur when these systems became active, i.e., during adolescence. This would seem to imply, however, that drug use would then be more closely associated with sexual, parenting, or pair-bonding emotions, which is debatable.

^19^Disulfiram is a partial example. It inhibits acetaldehyde dehydrogenase, which, in individuals who drink, leads to high levels of acetaldehyde, a toxic alcohol metabolite. The resulting unpleasant reaction deters drinking.
